# Force determination in lateral magnetic tweezers combined with TIRF microscopy†
†Electronic supplementary information (ESI) available. See DOI: 10.1039/c7nr07344e


**DOI:** 10.1039/c7nr07344e

**Published:** 2018-02-20

**Authors:** J. Madariaga-Marcos, S. Hormeño, C. L. Pastrana, G. L. M. Fisher, M. S. Dillingham, F. Moreno-Herrero

**Affiliations:** a Department of Macromolecular Structures , Centro Nacional de Biotecnología , Consejo Superior de Investigaciones Científicas , 28049 Cantoblanco , Madrid , Spain . Email: fernando.moreno@cnb.csic.es; b DNA:Protein Interactions Unit , School of Biochemistry , Biomedical Sciences Building , University of Bristol , Bristol , BS8 1TD , UK

## Abstract

Combining single-molecule techniques with fluorescence microscopy has attracted much interest because it allows the correlation of mechanical measurements with directly visualized DNA : protein interactions. In particular, its combination with total internal reflection fluorescence microscopy (TIRF) is advantageous because of the high signal-to-noise ratio this technique achieves. This, however, requires stretching long DNA molecules across the surface of a flow cell to maximize polymer exposure to the excitation light. In this work, we develop a module to laterally stretch DNA molecules at a constant force, which can be easily implemented in regular or combined magnetic tweezers (MT)–TIRF setups. The pulling module is further characterized in standard flow cells of different thicknesses and glass capillaries, using two types of micrometer size superparamagnetic beads, long DNA molecules, and a home-built device to rotate capillaries with mrad precision. The force range achieved by the magnetic pulling module was between 0.1 and 30 pN. A formalism for estimating forces in flow-stretched tethered beads is also proposed, and the results compared with those of lateral MT, demonstrating that lateral MT achieve higher forces with lower dispersion. Finally, we show the compatibility with TIRF microscopy and the parallelization of measurements by characterizing DNA binding by the centromere-binding protein ParB from *Bacillus subtilis*. Simultaneous MT pulling and fluorescence imaging demonstrate the non-specific binding of *Bs*ParB on DNA under conditions restrictive to condensation.

## Abstract 2

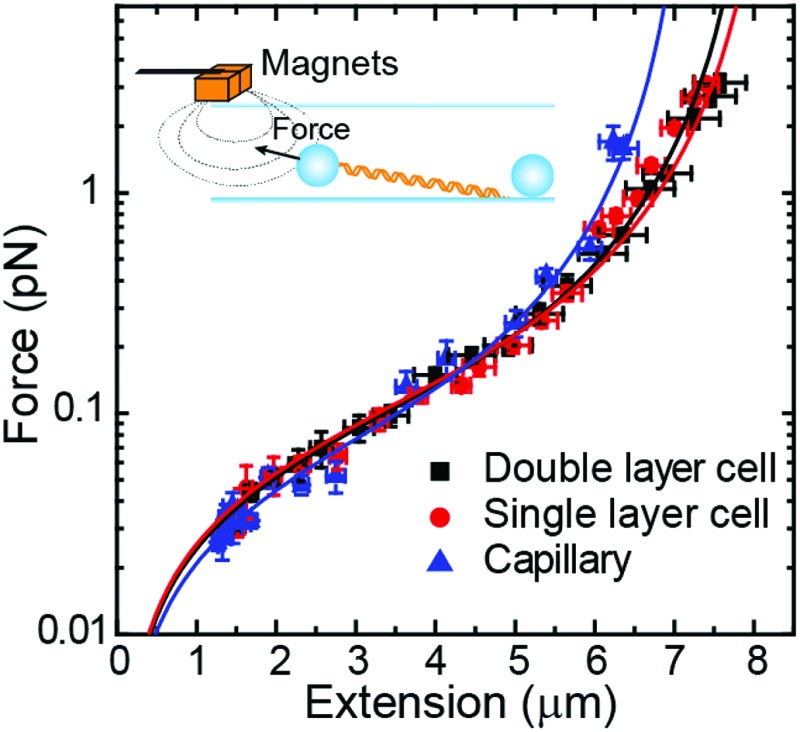
We have designed and calibrated a magnetic tweezers module to laterally stretch DNA molecules at a constant force, which can be incorporated into conventional magnetic tweezers. We demonstrate the combination of lateral magnetic tweezers with TIRF microscopy by characterizing DNA binding by ParB.

## 1. Introduction

In recent years, there has been increasing interest in combining force spectroscopy with fluorescence microscopy.^1^,^2^ These combined setups, built upon magnetic tweezers (MT), optical tweezers (OT), and atomic force microscopy (AFM), are powerful tools permitting the manipulation of individual molecules at the same time they are visualized. For example, DNA has been directly visualized with fluorescence microscopy using intercalating dyes during the mechanical disassembly of viruses by AFM,^3^ and proteins involved in DNA repair have been directly observed while their mechanical action on the DNA was probed with OT.^4^ Thus, these are useful techniques to couple the mechanical properties of biomolecules with DNA–protein interactions monitored in parallel.^5^

Experimental setups combining OT with epifluorescence/super-resolution or AFM with total internal reflection fluorescence (TIRF) microscopy have been reported in the literature,^6^–^11^ and are even commercially available in some particular cases. In contrast, a few studies have been reported on combinations of MT and fluorescence – in particular TIRF microscopy.^12^–^16^ The strength of combining these two techniques relies on the advantages they have separately. On the one hand, magnetic tweezers permit the simultaneous tracking of several individual (non)torsionally constrained DNA molecules anchored on the surface of a flow cell, while a force is applied in a controlled manner.^17^ On the other hand, TIRF microscopy exhibits a superior signal-to-noise ratio over other fluorescence-based techniques. TIRF microscopy relies on illuminating the sample with an incident angle higher than the critical angle, generating an evanescent field that only reaches a few hundreds of nanometres from the experimental surface. Hence, the excitation of fluorescent probes is limited to that volume.^18^,^19^ The drawback, however, is that, to fully exploit the advantages of TIRF microscopy, long DNA molecules need to be stretched across the surface of the flow cell. Methods to stretch DNA molecules across a glass surface include DNA combing^20^ and its variations to spread DNA fibers and chromosomes.^21^ DNA molecules can also be tethered between two defined locations on a glass surface generating the so-called DNA curtains.^22^ However, in both methodologies the force applied to the DNA molecules/fibers cannot be easily inferred. One of the most widespread manners of visualizing fluorescent DNA molecules on a surface at the same time they are sensing a force is to stretch them under a continuous flow.^2^,^23^,^24^ Note however that the force applied to flow-stretched DNA is not constant along the DNA molecule, being larger at the anchoring point than at the tip, and thus it is also difficult to estimate.^25^ Here, we argue that a way to have accurate control of the pulling force in combined systems with TIRF microscopy is by using lateral magnetic pulling.

Lateral magnetic pulling (perpendicular to the optical axis) of DNA molecules has been already reported, using multiple strategies to tether the magnetic beads,^16^,^26^–^31^ but a thorough analysis of force calculation is still missing. Pioneering studies used the angle described by a tethered bead subjected to simultaneous lateral pulling and perpendicular flow stretching to infer the applied force.^31^ Other authors tethered a DNA hairpin to a round capillary that was subsequently unzipped providing a fingerprint for force estimation.^27^ However, the use of a round capillary made it difficult to measure anchoring points on the surface, possibly leading to underestimation of the extensions. More recently, the magnetic force was calibrated based on the Stokes drag experienced by magnetic beads in glycerol, and checked using a Gauss meter and the known magnetization of the beads.^32^ In other work, a duplex DNA molecule was tethered between two beads; one was held by a micropipette while the other one was laterally pulled by a magnet.^26^,^28^ This is advantageous for proper extension determination since both beads could be placed in the same focal plane, but lacks the parallelization that is desirable when using MT in single-molecule experiments. Recently, lateral MT combined with fluorescence has been implemented by some groups,^16^,^29^,^30^ but with limited reference to force determination.

In this work, we describe a module to apply forces to laterally stretch DNA which could be easily incorporated into different MT setups. We describe a methodology to determine the force exerted on laterally-pulled DNA molecules based on thermal fluctuations of the bead, as used in standard vertical MT.^31^,^33^ We characterized both cover-glass flow chambers and glass capillaries, and discuss the advantages and drawbacks of each fluidic system. For the sake of comparison, we have also measured drag forces on flow stretched DNA molecules attached to beads. Finally, our lateral pulling device was combined with TIRF microscopy. This setup gives access to experiments where one can simultaneously visualize DNA binding proteins under controlled stretching forces. We have applied this combined setup to study the DNA binding and condensation activity of ParB, a component of the ParABS partitioning system, involved in bacterial chromosome segregation and condensation. Our work provides a guide to implement lateral magnetic tweezers compatible with TIRF microscopy and a reference of the force magnitude that can be applied.

## 2. Results and discussion

### 2.1. Implementation of lateral magnetic tweezers

**Fig. 1:**
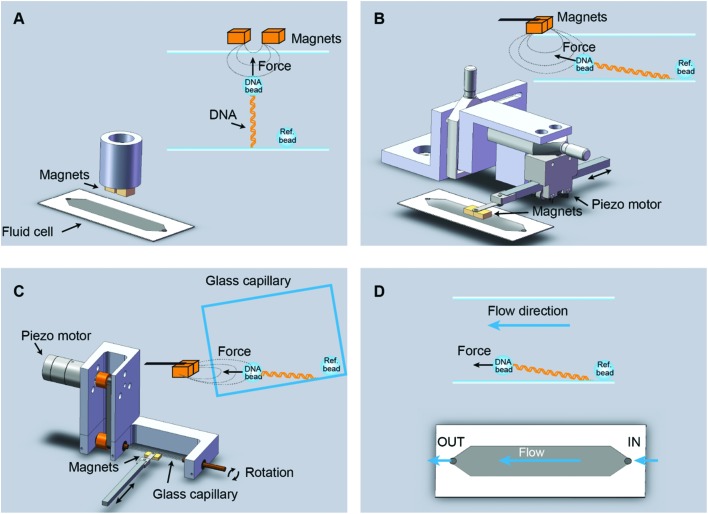
Different setups for vertical and lateral pulling of DNA. (A) Conventional vertical magnetic tweezers. Magnets approach from the top to a regular flow cell and are aligned with the optical path. (B) Lateral magnetic tweezers based on a regular flow cell. In this configuration, magnets approach from one side of the cell, stretching the DNA tethers along the surface. Magnets are coupled to a home-built module that allows their positioning and movement with sub-micron precision (Table S1†). (C) Lateral magnetic tweezers in a glass square capillary. A square capillary is held by a home-made module that also allows rotation with mrad (0.1°) precision. The capillary was tilted ∼5° and this facilitated the alignment of DNA tethers to the horizontal plane. This setup is also compatible with vertical pulling as performed in panel A. (D) DNA flow-stretch experiments. A DNA tether is stretched under flow, in the absence of magnetic force. The drag force stretches the molecule across the surface.

In vertical MT, a pair of permanent magnets aligned with the optical axis pull superparamagnetic beads tethered to the flat surface of a flow cell by DNA molecules (Fig. 1A). Flow cells are commonly made with a paraffin wax film (parafilm) sandwiched between two glass coverslips. The force range depends on the magnet-bead distance, which is limited by the thickness of the flow cell, and on the bead size. For instance, in our vertical MT, the combination of two-parafilm layer cells (200 μm thickness) and 1 μm beads achieve 3–4 pN of maximum force. The position of the bead at different focal planes can be tracked by optical microscopy. The determination of the extension relies on measuring the distance between a tethered bead and a reference bead, fixed on the surface. This geometry assumes that DNA binds along the central axis of the bead, which rarely occurs. However, for most applications one is interested in relative changes in extensions and these can be accurately determined with a precision of a few nanometres.^34^ When combined with fluorescence the vertical magnet configuration is not convenient because molecules are stretched along the axis of visualization. In order to visualize proteins interacting with the DNA, the polymer should be extended across the surface, perpendicular to the optical axis, and this can be done by lateral magnetic pulling.

We custom-built and implemented a lateral pulling module using a pair of permanent magnets (Q-05-05-02-G, Supermagnete) connected to a linear piezoelectric motor (Piezomotor) (Fig. 1B, see Table S1† for a list of components and Fig. S1–S3† for technical drawings). This module allowed positioning of the magnets in the optical axis, just above the flow cell with micrometer precision using translation stages (Newport). The piezoelectric motor drives a plastic rod with the magnets at the end that can be moved over a 15 mm range. The motor incorporates an encoder that provides a measurement of the position of the magnet and this enables closed-loop operation with sub-micrometer precision. Custom-scripts were implemented to ease the calibration procedure and to allow complete automation of magnet positioning (see below). In the lateral MT depicted in Fig. 1B, the flow cell is identical to the one used in the conventional vertical magnet configuration. DNA molecules are thus tethered as in vertical MT, but instead, pulled laterally using a pair of magnets that arrive from one side of the flow cell. This procedure allows stretching of DNA molecules in the visualization plane. The lateral pulling module can be easily implemented in an already working MT setup, making minor modifications to the sample cell holder.

Lateral pulling has been reported before in setups in which the sample is introduced in a square glass capillary and pulled laterally from one side.^29^,^30^ The advantage of using capillaries is that minor sample volumes are needed, and that by rotating the capillary the DNA molecule can be oriented perpendicular to the visualization axis.^35^ We have built a device to hold and rotate glass capillaries for lateral magnetic tweezers (Fig. 1C, see Table S2† for a list of components and Fig. S4 and S5† for technical drawings). The device comprises a rotary motor (Piezomotor) connected to a glass capillary tube (Vitrotubes) by a timing belt and pulleys as shown in Fig. 1C. The motor also incorporates an encoder enabling closed-loop operation, permitting us to control the rotation angle with a precision of 1 mrad. This capillary module allows the user to subtly rotate the capillary to either ensure a perfect surface flatness suitable for vertical pulling or to tilt the surface to ensure proper alignment of the DNA in the *x*–*y* plane when pulled laterally.

An alternative way to force-stretch DNA molecules across a surface is by applying a constant flow.^2^,^24^ Often this methodology is combined with fluorescence to visualize DNA molecules while they are being stretched by the flow. In flow-stretch experiments, the free DNA end experiences wide fluctuations due to the low drag force applied at the tip and this impedes a precise measurement of the extension of the DNA and the estimation of the average applied force. In order to determine the end position of the DNA we performed flow-stretch experiments on tethered DNA molecules with a micrometer size bead attached at the distal end of the DNA (Fig. 1D). The use of a bead at the DNA end allowed the measurement of the extension with a few nm precision. In addition, this approach allowed us to exert constant and larger forces along the DNA tether compared to DNA flow-stretching experiments.^25^ Using these data and Stokes’ law we estimated the velocity of the flow in the vicinity of the bead (see below).

### 2.2. Determination of the pulling force in lateral magnetic tweezers

In magnetic tweezers, forces acting on a tethered bead are calculated by measuring the Brownian fluctuations of the bead and the extension of the DNA molecule.^33^ This force is computed using eqn (1), where *k*_B_ is the Boltzmann constant, *T* is the temperature, *l* is the extension of the molecule, and *dy*^2^ is the variance of the fluctuations of the bead in the transverse direction, perpendicular to the optical axis.1
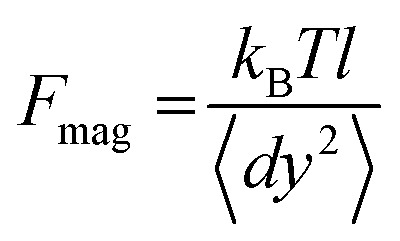



Bead coordinates are inferred from its diffraction rings in an out-of-focus optical image. The in-plane coordinates (*xy*) of the bead are obtained from cross-correlation analysis of an image with its mirrored image and the vertical position (*z*) by comparing diffraction ring patterns from a calibration look-up-table taken on a fixed reference bead.^34^,^36^ Fluctuations are analyzed in the frequency domain using power spectral density analysis.^37^ Our software also includes corrections for camera blurring and aliasing artifacts, which arise from finite camera acquisition frequencies and shutter time.^38^,^39^

**Fig. 2:**
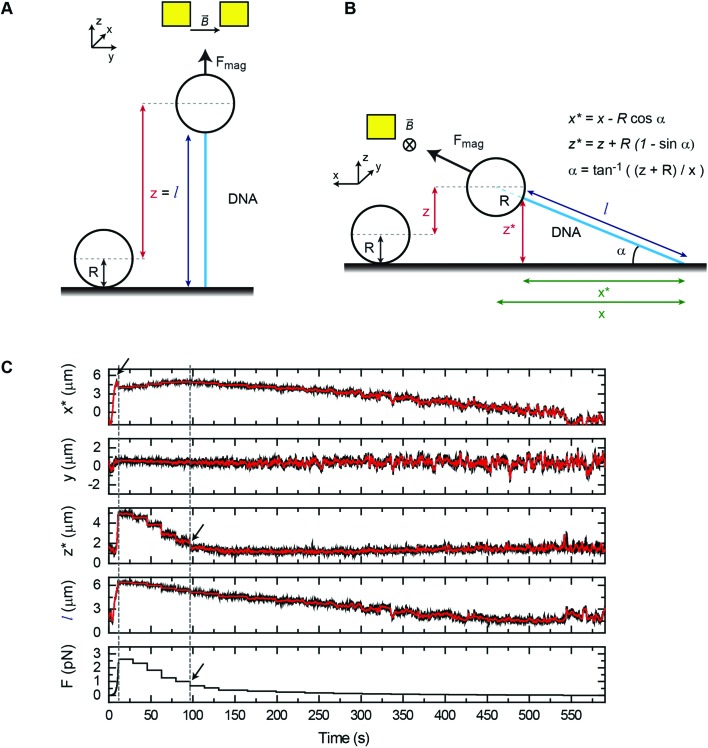
Measurement of the force in lateral magnetic tweezers in regular flow cells. (A) A cartoon of the geometric representation of extension (*l*) measurements in vertical pulling, considering the DNA molecule is attached to the lowest part of the bead. (B) A cartoon of the geometric representation of extension measurements in lateral pulling. The extension is computed as 
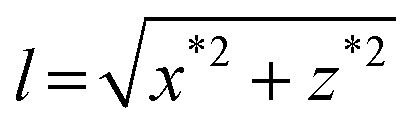
. This assumes that the DNA is attached analogously to vertical pulling. (C) Position coordinates (*x*, *y*, *z*) and extension (*l*) of a DNA molecule measured in a lateral pulling cycle, where the force is first suddenly increased (by moving the magnet to the closest position) (*t* = 20 s) and then decreased, in a stepwise manner. This produced a fast increase of the *x* signal and a small peak (arrow) that occurs just before the lift-off of the bead from the surface (left dashed line). The bead rests again at the surface beyond *t* = 95 s where the *z* measurement is close to zero (black arrow, and right dashed line), and the *x* coordinate recovers its maximum value.

We have first considered the simplest scenario where the DNA is attached to the central axis of the bead at its lowest part (Fig. 2A). In this case and for vertical tweezers, the DNA extension coincides with the distance between DNA- and Ref-bead centers, which is the *z* value that the magnetic tweezers setup provides. When pulling laterally (Fig. 2B), it is advantageous to keep the same magnetic field orientation as in vertical pulling because the axis of fluctuations (*y*) is maintained in both vertical and lateral pulling configurations. In this sense, the same acquisition and software analysis can be used to calculate the force using eqn (1). The extension of the DNA, however, has to be calculated considering *x* and *z* coordinates of the bead. In standard flow cells made of two cover slides, the DNA end coordinates at the bead (x* and z*) can be determined using eqn (2)–(4):

2 x* = *x* – *R* cos *α*

3 z* = *z* + *R*(1 – sin *α*)

4 *α* = tan^–1^ ((*z* + *R*)/*x*)

where *R* is the bead radius and *α* is the angle formed by the DNA molecule and the surface (Fig. 2B). A precise measurement of *x* needs to consider the attachment point of the DNA on the surface. This is determined as the centre of the projected circle described by a tethered bead in the *xy* plane, while magnets are rotated in the vertical configuration. The maintenance of the same magnetic field orientation in both vertical and lateral magnetic tweezers avoids changes in the mean *y* position of the bead when pulled laterally, and reduces the extension of the DNA to 
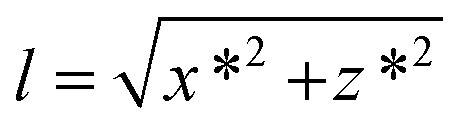
.

Typical time courses of a lateral pulling experiment in the conventional cover-glass cell are shown in Fig. 2C. The force was quickly raised by approaching the lateral magnet to the central part of the flow cell causing the extension of the DNA to reach a maximum value (see the blue arrow in x* data) followed by the lift-off of the bead (see z* data), which necessarily made the x* coordinate to decrease. As the force is reduced by moving the magnet away from the bead, the vertical coordinate reduced and the x* coordinate recovered its maximum value. Note that the transverse coordinate *y* remains around zero for the complete cycle of extension consistent with both vertical and horizontal magnetic fields having identical orientation. Molecules were fully extended on the surface at around 1 pN force (see the black arrow in force data) and a maximum force of 3–4 pN was obtained, very similar to that achieved with the standard vertical configuration.

A more realistic scenario considers that DNA attaches to an off-center point from the bead vertical axis (Fig. S6A†). In the vertical pulling configuration, the extension of the DNA molecule (*l*) is now corrected by *z*_corr_, a factor dependent on the attachment point of the DNA on the bead and on the applied force.^40^ In lateral tweezers (Fig. S6B†), the off-center attachment also adds a correction to the coordinates of the DNA end at the bead (see the ESI† for a detailed mathematical description).

**Fig. 3:**
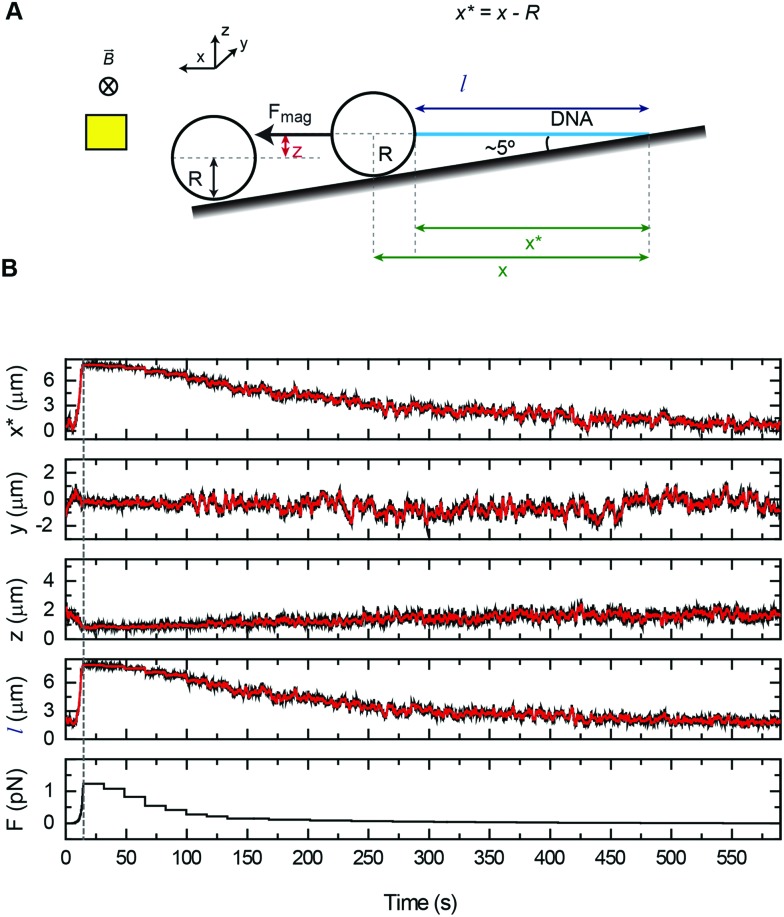
Measurement of the lateral force in square glass capillaries. (A) A cartoon of the geometric representation of extension measurements in lateral pulling when using a square glass capillary tube. When the capillary is tilted ∼5°, the extension is simply *l* = *x* – *R*. (B) Position coordinates (*x*, *y*, *z*) and extension (*l*) of a DNA molecule measured in a lateral pulling cycle in a square capillary where the force is increased by approaching the magnet to the closest position (*t* = 20 s) and then decreased stepwise. Note that in glass capillaries, there is no peak in *x* as the bead is permanently in contact with the surface, and *z* measurement is close to zero throughout the entire measurement.

We have also explored the lateral pulling geometry using square glass capillaries. Our home-built device allows tilting the capillary to extend the DNA molecule along the surface pulling from one side (Fig. 3A). The tilt was adjusted to maintain the *z* coordinate of the bead roughly constant during the pulling cycle and it was about 5°. This makes the extension of the DNA to be essentially the *x* coordinate minus the radius of the bead. Since there is no lift-off of the bead in this case, the DNA molecule can be fully extended along the surface up to the maximum applied force (Fig. 3B). In this configuration, similar corrections due to off-center bead attachments are applicable to the calculation of the extension (see the ESI†).

### 2.3. Characterization of pulling forces for different magnet configurations and bead sizes

The lateral pulling methodology described above was applied to *λ*/2 molecules (24.5 kbp long) tethered in single (100 μm thickness) and double (200 μm) parafilm layered flow cells and in square glass capillaries to explore different cell configurations and available ranges of forces. We employed magnetic beads of 1 μm and 2.8 μm diameter sizes and compared the maximum applied forces and force–extension curves obtained from both vertical and lateral magnet configurations.

The force curve was exponentially dependent on the magnet distance as previously reported for all pulling configurations and bead sizes (Fig. S7†).^36^ The highest forces were achieved using single layer cells in the vertical configuration (Table 1). We measured forces up to 4.7 and 30 pN with 1 μm and 2.8 μm beads, respectively. The lateral configuration using single parafilm layer cells achieved lower maximum forces, and these were reduced to 0.8 pN and 4 pN for 1 μm and 2.8 μm beads at the lift-off point, where the DNA molecule stands up from the surface (see the arrow in the force panel in Fig. 2C, and Fig. S8† for clarification). In each case, the glass capillary configuration achieved lower maximum forces due to the thickness of the capillary walls and the dimensions of the channel, resulting in larger magnet-bead distances.

**Table 1:** The maximum force achieved for different setups and bead sizes. Errors are the standard deviation

Bead size [μm]	Cell thickness [parafilm layers]	Magnet configuration	No of DNA molecules	Maximum force (maximum lateral force before lift-off) [pN]
1	2	Vertical	12	3.5 ± 0.5
		Lateral	10	3.1 ± 0.9 (0.6 ± 0.1)
	1	Vertical	12	4.7 ± 0.8
		Lateral	18	3.1 ± 0.7 (0.8 ± 0.2)
	Capillary	Vertical	12	1.4 ± 0.3
		Lateral	9	1.7 ± 0.7
2.8	2	Vertical	17	26 ± 9
		Lateral	19	21 ± 4 (6 ± 3)
	1	Vertical	13	30 ± 7
		Lateral	18	20 ± 7 (4 ± 2)
	Capillary	Vertical	16	9 ± 2
		Lateral	14	19 ± 12

**Fig. 4:**
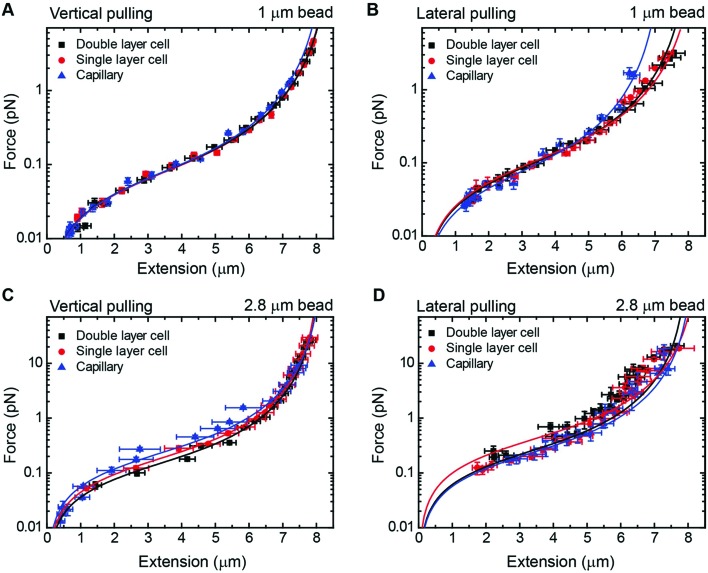
Force extension curves of DNA for each magnet configuration and bead size. (A) A vertical magnet configuration and 1 μm beads. (B) A lateral magnet configuration and 1 μm beads. (C) A vertical magnet configuration and 2.8 μm beads. (D) A lateral magnet configuration and 2.8 μm beads. Data were obtained for *λ*/2 DNA molecules and from flow cells of one or two layers of parafilm or from glass capillaries. Force–extension data were fitted to the worm-like chain model (solid line). Fitting parameters are shown in Table 2. Error bars are the standard error of the mean.

As a proof of principle of the calibration procedure, we next compared force–extension data obtained from vertical and lateral magnetic tweezers for different cell configurations and bead sizes (Fig. 4). For a given set of magnet positions we determined the extension of the molecule following the procedures described above, and from that value the applied force was calculated (eqn (1)). Data were fitted to the WLC model with the corrections given by Bouchiat *et al*.^41^ to obtain contour (*L*) and persistence (*P*) lengths (Table 2).

**Table 2:** Worm-like-chain model parameters from fittings of DNA force–extension curves for different setups and bead sizes. Errors are the standard deviation

Bead size [μm]	Cell thickness [parafilm layers]	Magnet configuration	No of DNA molecules	Persistence length (*P*) [nm]	Contour length (*L*) [μm]
1	1	Vertical	12	39 ± 1	8.4 ± 0.9
		Lateral	18	32 ± 2	8.5 ± 0.7
	2	Vertical	12	40 ± 2	8.1 ± 0.9
		Lateral	10	29 ± 1	8.4 ± 1.1
	Capillary	Vertical	12	40 ± 1	8.1 ± 1.1
		Lateral	9	41 ± 2	7.4 ± 1.0
2.8	1	Vertical	13	14 ± 1	8.4 ± 0.3
		Lateral	18	17 ± 1	8.0 ± 1.4
	2	Vertical	17	8 ± 1	8.3 ± 0.3
		Lateral	19	21 ± 1	8.3 ± 0.7
	Capillary	Vertical	16	13 ± 1	8.3 ± 0.5
		Lateral	14	14 ± 1	7.9 ± 0.8

Data obtained from the vertical magnet configuration using 1 μm beads (Fig. 4A) showed little variability of values of persistence length and contour length. We measured *P* = 39 ± 1 nm (*n* = 12, single layer cell), *P* = 40 ± 2 (*n* = 12, double layer cell), and *P* = 40 ± 1 (*n* = 12, capillary) (errors from fitting the WLC to the average force–extension curve). These values of *P* were consistent with previously reported values taken in the same experimental buffer.^42^ Contour length values were *L* = 8.3 ± 0.9 μm, *L* = 8.2 ± 0.9 μm, and *L* = 8.1 ± 1.1 μm, for single cell, double cell, and capillary, respectively (errors are the standard deviation of the mean *L* obtained from individual force–extension curves). The measured *L* was consistent with the length expected for a 24.5 kbp long DNA.

Lateral pulling data for 1 μm beads showed larger variability in both extension and force (Fig. 4B). In this case, surface interactions are likely to dump the magnitude of transversal fluctuations of the bead due to friction, resulting in overestimation of the force and larger variability of the data. This is particularly relevant at low forces in the case of cover glass based cells. Consistent with this idea, the capillary data at high forces (blue triangles) were above the measured forces in standard cells, where the bead is out of surface contact at high force (black squares and red circles). Remarkably, we found values of persistence and contour lengths in agreement within the experimental error to those measured with the vertical magnet configuration (Table 2). Thus, the lateral pulling configuration using capillaries is recommended if precise mechanical measurements of the tethered molecules are required. The correction in *z* due to off-center attachments at maximum force (≈4 pN) was only of 0.6% of the expected extension of *λ*/2 DNA molecules at that force. Therefore, the use of a simplified model to estimate extensions neglecting off-center attachments was justified for 1 μm beads.

In the case of 2.8 μm beads and vertical magnet configuration (Fig. 4C), force–extension curves nicely overlapped but the fit to the WLC gave a value for the persistence length much lower than expected (Fig. S9†). This deviation from the WLC curve has been reported before^40^ and it was attributed to the off-center attachment of the DNA molecule to the bead. Indeed, additional measurements obtained from a double layer cell and analyzed taking into account the geometry of the system and the anchoring point of the DNA at the bead substantially improved the force extension fitting parameters (Fig. S10†). These measurements considered values of *z*_corr_ taken at different forces, which involved rotations of the vertical magnet to measure the off-center position of the DNA in the bead.

The case of large beads and lateral pulling (Fig. 4D) showed the cumulative detrimental effects of the previous cases. In general, we observed a much larger variability of the data, likely due to the friction of the bead with the surface, but also due to the additional effects of using large beads and unavoidable off-center attachments. Direct measurements of the corrections in the extension due to the off-center attachment of the DNA to the bead in the lateral configuration were not possible because of the restricted objective-magnet geometry.

### 2.4. DNA flow stretching achieves lower force values and results in noisier measurements

An extended method to study DNA–protein interactions at the single molecule level using fluorescence microscopy is to stretch it under flow.^23^,^43^ Although this technique is mainly qualitative in terms of force, there have been attempts based on labelling specific sites along the duplex to quantitatively estimate the force exerted on the DNA molecule.^25^ However, in flow-stretched DNA, the force is not uniform along the DNA molecule being larger at the anchoring point and lower at the free DNA end. This makes difficult to precisely determine the extension of the tether and to correlate mechanical features with fluorescence events in a quantitative manner.

**Fig. 5:**
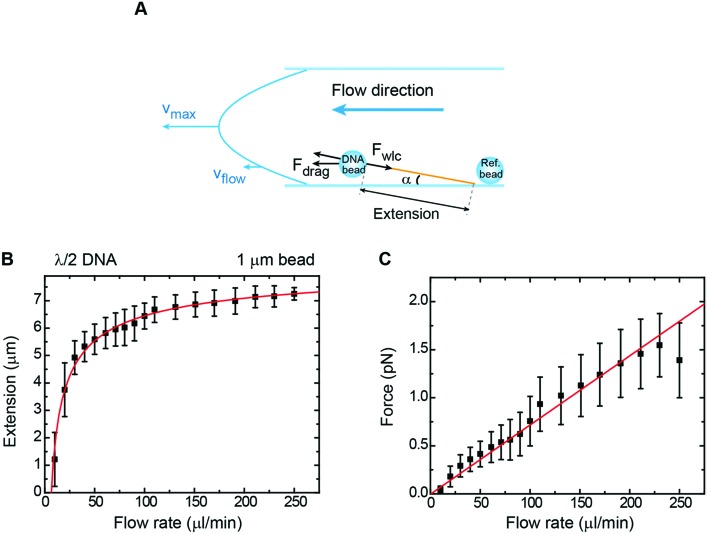
Characterization of forces in flow-stretch experiments. (A) A cartoon depicting geometry on a flow-stretch experiment. Under laminar flow conditions, the velocity profile is parabolic. (B) Mean DNA extension as a function of flow rate in a flow stretch experiment using 1 μm beads and *λ*/2 DNA molecules. The solid line is the fit to eqn (8) with *P* = 40 nm as a fixed parameter, obtaining *L* = 8.6 μm. (C) Mean force as a function of flow rate for the same data set. The force was determined from a calibration force–extension curve obtained from the vertical configuration in the absence of flow. The force increases linearly up to a maximum value of ∼1.5 pN, in accordance with eqn (9). Error bars in B and C are the standard deviation of the mean from measurements of multiple beads.

An alternative way to stretch DNA by drag consists of attaching a bead to the DNA end and controlling an applied force by using a constant flow (Fig. 5A).^44^ The use of a bead at the end of the DNA allowed us to precisely measure the extension of the tether by tracking the bead and considering the anchoring point, as determined using the rotation procedure described above. Extension *versus* flow data can be correlated with the applied force using a previously-taken force–extension curve performed with the vertical magnet configuration. This allowed us to correlate the mean extension of a particular tether stretched laterally by the drag force produced by a certain flow rate (*Q*). Experiments were performed with 1 μm beads and *λ*/2 DNA molecules in a regular two-parafilm layer flow cell. Flow rates were set using a computer-controlled syringe pump (Nemesys) up to 250 μl min^–1^. At this maximum flow rate the molecule extended up to 93% of its crystallographic length (Fig. 5B) and forces estimated from the WLC model (*F*_WLC_) increased linearly with the flow rate up to 1.5 pN (Fig. 5C).

From our data it is possible to estimate the velocity of the flow in the vicinity of the bead. Our system is under laminar flow conditions (*Re* ∼10^–3^, see the ESI†) and therefore, the bead experiences a drag force given by Stokes’ law:

5 *F*_drag_ = 6π*Rηv*_flow_

where *v*_flow_ is the linear velocity of the flow in the vicinity of the bead, *R* is the radius of the bead and *η* is the viscosity of the fluid. As expected, the linear trend given by Stokes’ law was experimentally observed (Fig. 5B).

The linear velocity of the flow can be expressed as a fraction of the maximum velocity at the centre of the channel (eqn (6)), which is defined as 
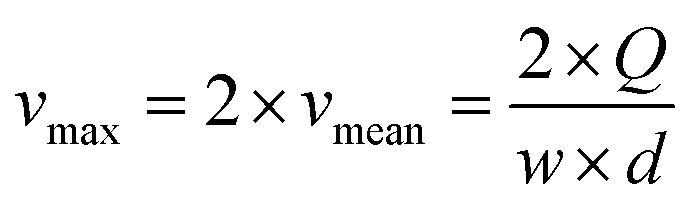
 ,^45^,^46^ where *d* and *w* correspond to the channel height and width, respectively. In our case, *d* ≈ 200 μm and *w* ≈ 7 mm, yielding a cross section of the cell of 1.4 mm^2^.6
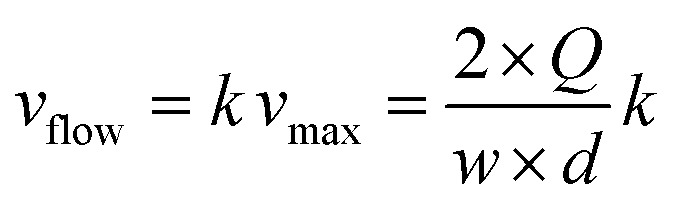



Note that the viscosity, *η*, should be corrected because the radius of the bead is comparable to the distance of the bead to the surface following eqn (7).^47^ At *z* ≈ 1 μm and 1 μm beads we obtain η* = 1.6*η*.7
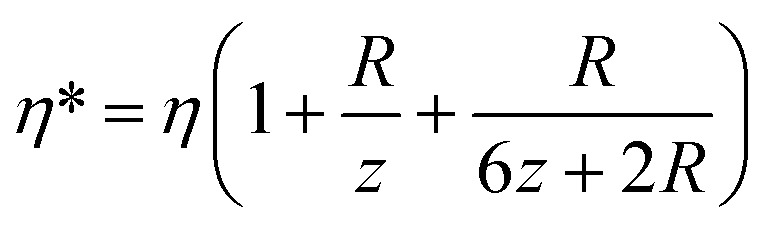



At equilibrium, *F*_WLC_ = *F*_drag_/cos *α* ≈ *F*_drag_, for *α* → 0. We can then estimate the linear velocity of the flow in the proximity of the bead, by fitting eqn (8) and (9) to the extension data (Fig. 5B) and the force data (Fig. 5C).8
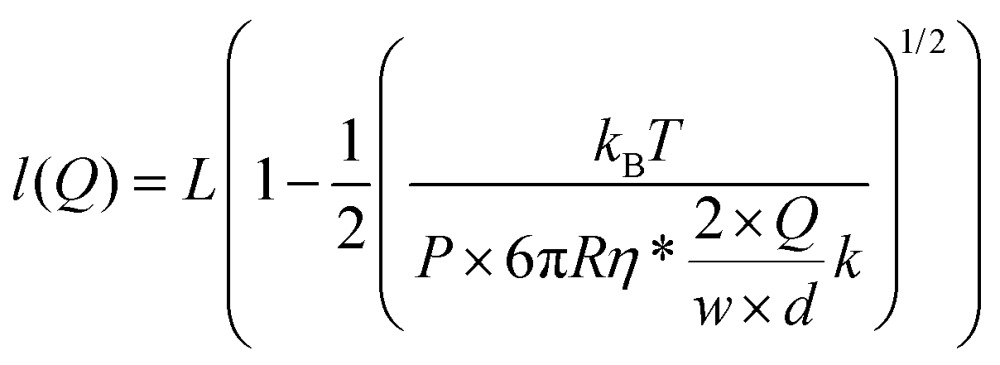

9
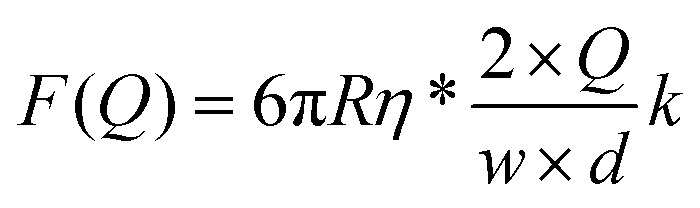



Assuming a persistence length of 40 nm, we obtained a contour length of *L* = 8.6 μm, which is very close to the expected crystallographic length of the molecule, and *v*_flow_ = 0.011*v*_max_ (1.1% of *v*_max_) from the fitting to the extension data, and *v*_flow_ = 0.02*v*_max_ (2.0% of *v*_max_) from the fitting to the force data.

The linear velocity of the flow at a distance from the surface can also be calculated by considering a uniform laminar flow through a practically infinite channel, which can be approximated by the same type of flow through a circular tube (Fig. 5A).^45^,^46^
10
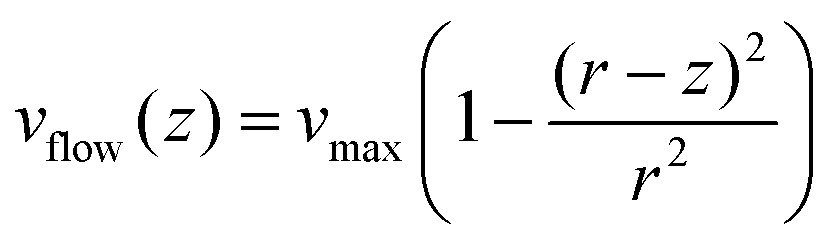
where *r* is the equivalent radius defined as *r* = (*d* × *w*)/(*d* + *w*) and *z* is the distance from the surface. Note that eqn (10) is independent of the viscosity of the fluid.

To obtain the mean velocity acting on the bead, we integrated the parabolic velocity profile over the diameter of the bead, and divided it by the diameter itself (see the ESI†). This calculation resulted in a velocity of 1% of *v*_max_ in good agreement with our experimental data. Nevertheless, it must be taken into account that Stokes’ law does not account for boundary (turbulence) effects that are probably affecting the bead in the vicinity of the surface. In this case, correcting the viscosity near the surface may not be enough to estimate a proper velocity.

Our analysis from drag experiments provided a value of the maximum force of 1.5 ± 0.2 pN, similar to other experimental approaches based on flow-stretched DNA.^25^ Larger flow velocities near surface and hence larger forces on the DNA could be achieved by reducing the dimensions of the flow cell. Our approach assumes that the drag force acting on the DNA is negligible because the microscopic bead is massive compared to the stretched DNA. Therefore, we have considered that the force is applied only at the DNA end and constant along the tether. The magnitude of forces measured in our bead-based flow-stretch experiments was below the forces applied by the magnets in any of our MT configurations using double-layer cells. Moreover, the forces measured in flow-stretch experiments showed larger dispersion. These observations illustrate the advantages of using magnets to laterally stretch DNA in the standard flow cells of large inner volume employed in this work.

### 2.5 Simultaneous MT and TIRF measurements demonstrate DNA binding and condensation by ParB

*Bacillus subtilis* ParB is a centromere-binding protein involved in bacterial chromosome segregation. It specifically binds to the centromere-like DNA sequence *parS*, but it also has a poorly characterized non-specific binding mode responsible for the association with DNA for several kilobases around *parS* sites.^48^,^49^ Because there are only around 20 ParB dimers per *parS* sequence in the cell, this “spreading” is thought to require the formation of three-dimensional ParB networks.^23^,^50^,^51^ However, the mechanism that underlies the formation of intermolecular bridges between ParB molecules is largely unknown. We have previously shown that the non-specific interaction of *Bs*ParB with DNA leads to condensation using vertical MT at permissive forces below 1 pN.^52^ Nonetheless, MT experiments do not allow the correlation of protein binding and condensation as a function of the force. Flow-stretch experiments combined with TIRF microscopy have also visualized ParB binding but the force applied by the flow could not prevent condensation.^23^ Moreover, these experiments present a flow-induced artefact of condensation from the DNA end because the force exerted on the DNA by the flow is not uniform.

**Fig. 6:**
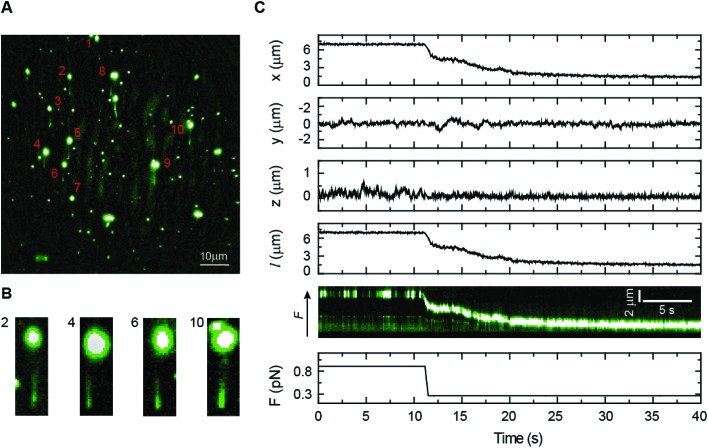
Combined lateral MT and TIRF microscopy. (A) A TIRF image showing multiple laterally-stretched DNA molecules covered with ParB^AF^. Protein binding illuminates the DNA tethers. (B) Details of several DNA molecules, where the DNA molecule is visible. (C) Simultaneous bead tracking and fluorescence imaging for a single DNA molecule condensed by ParB^AF^. The force was dropped from 0.9 to 0.3 pN to permit condensation. The reduction in extension coming from condensation was correlated with the fluorescence kymograph, where the bead is dragged towards the anchoring point of the molecule. At high force, fluctuations of the DNA molecule means that the bead occasionally exits the evanescent wave, and therefore emits no fluorescence.

We directly monitored *Bs*ParB binding to single DNA molecules by coupling our lateral pulling module to a home-built MT–TIRF microscope setup (see Experimental section). Combining these techniques, we were able to prevent DNA condensation by ParB for the first time, while studying the binding of the protein. We used a fluorescent variant of ParB labelled with Alexa Fluor 488 (ParB^AF^), functional for both specific and non-specific DNA binding *in vitro* (data not shown), to identify protein binding by fluorescence. As shown in Fig. 6A, several DNA molecules are laterally pulled, and a DNA–ParB^AF^ filament is clearly visible under TIRF microscopy. In this particular example, about ten DNA molecules could be observed in the same field of view, demonstrating the parallelization capabilities of our instrument. Note, however, that none of them had the entire DNA filament visible. As would be expected, due to the limited excitation volume produced by TIRF and the tilting of the DNA (Fig. 2C), the DNA fragment close to the bead remains invisible under the evanescent wave. The visible length also depends on the anchoring point on the bead, resulting in certain variability in visible lengths from molecule to molecule. Details of several DNA molecules are shown in Fig. 6B. The visible region of the filament could be extended by increasing the length of the DNA, the intensity of the laser, and/or changing the incident angle of the beam to increase the penetration depth of the evanescent field.^53^ Our experimental conditions, however, require a high concentration of fluorescent protein and the illumination region must be restricted to a few hundreds of nm to minimize background illumination.

To correlate the DNA extension data from MT and the fluorescence signal from ParB^AF^, the force was reduced using lateral magnets to allow ParB^AF^ to condense the DNA. A sample curve can be seen in Fig. 6C, where the force is reduced from 0.9 to 0.3 pN to allow condensation of the DNA by the protein. The bead tracking from MT correlates well with the fluorescence kymograph, demonstrating the capabilities of our laterally pulling module to be coupled with fluorescence microscopy. The fluorescence data indicate that condensation is not dependent on the formation of condensation clusters, but is rather a uniform process occurring along the full length of the DNA molecule. This combined setup allowed us to visualize for the first time the binding of ParB to DNA at the single molecule level while applying a constant and controlled non-permissive force for condensation.

## 3. Conclusion

In this work, we introduced a lateral pulling approach based on MT and a device to hold and rotate glass capillaries to ensure genuine horizontal pulling of DNA. We have proved our approach to be simple to implement and compatible with conventional MT, requiring minor design modifications. This module allows applying well-controlled constant forces to tethered DNA molecules, stretching them parallel to the surface and thus will allow direct visualization of DNA and/or DNA–protein interactions. Furthermore, we have tested lateral MT in different flow cell configurations using commercially available superparamagnetic beads. Lateral MT can be force-calibrated based on the method used in vertical MT, disregarding corrections arising from off-centre attachments, with a dispersion of less than 5%. The calibration procedure was validated with force–extension curves in different cells and bead combinations, showing a good range of agreement. Higher dispersion in lateral MT forces was attributed to surface–bead interactions. The measurement of lower persistence lengths in the case of 2.8 μm diameter beads was shown to result from off-centre attachments. Capillaries allowed us to apply maximum horizontal forces compared to the forces obtained from regular cells before bead lift-off. Single-layer cells enabled maximum vertical forces up to 30 pN. By monitoring single DNA extension and using individual force extension curves, we were able to estimate forces in a bead pulled in flow-stretch experiments, showing that measured forces were lower and more dispersive than the ones in lateral MT. The strength of our lateral pulling device also relies on its combination with TIRF microscopy. We have coupled our lateral MT to a fluorescence microscope, and have demonstrated its use by studying the DNA binding activity of *B. subtilis* ParB. Our results open the possibility to study and visualize ParB binding at non-permissive forces for condensation and to investigate the processes of protein nucleation and exchange (a subject of future work).

## 4. Experimental section

### 4.1. Construction of a lateral magnetic tweezers setup

The Lateral magnetic tweezers (Fig. 1B) consist of a pair of permanent magnets (Q-05-05-02-G, Supermagnete) connected to a linear motor (Piezomotor) that can be controlled by a PC encoder (Piezomotor). This lateral magnet is incorporated into an already running vertical magnetic tweezers setup assembled as described previously.^17^ Vertical magnetic tweezers used two magnets (W-05-N50-G, Supermagnete) in horizontal configuration held in an iron yoke leaving a gap of 2 mm between them. For standard single- or double-layer flow cells, the origin position of the lateral magnet was set as follows. The vertical origin (*z* = 0) was set to the point where magnets touch the sample cell, plus a small offset for safety (0.2 mm). The horizontal origin was set to the point in which the vertical magnet fully covers the microscope objective, as determined from the optical image. These manual alignments of the lateral magnet resulted in slightly larger variability between flow cells, compared to the vertical magnet configuration. For the capillary, the zero position of the magnets was defined by slight contact with the capillary.

### 4.2. Combined lateral magnetic tweezers with TIRF microscopy setups

A 488 nm laser source (Vortran Stradus) was focused on the back focal plane of a high numerical aperture objective (Olympus UAPON TIRF 100×). We used two separate detectors to visualize the emission of the fluorophores in the sample and the magnetic beads; an EM-CCD temperature-controlled camera (Andor Ixon Ultra 897) and a CCD camera (Pulnix 6710CL) for bright-field video microscopy. The fluorescence and bright-field signals were separated using a dichroic mirror, which permits using a single optical path for both detectors (Fig. S11†).

### 4.3. Construction and functionalization of flow cells and capillaries

Coverslips (Menzel-Gläser, #1) were cleaned by 30 minutes of sonication in acetone and 30 minutes in isopropanol, and dried using compressed air. A 1 : 120 dilution from the stock of 1 μm or 2.8 μm sized beads (Dynabeads, MyOne streptavidin, Invitrogen) in ethanol was spread on the bottom glass surface (3 μl) before it was heated up for 3 minutes at 120 °C. The surface was then coated with 1% polystyrene dissolved in toluene. The top cover glass contained two holes drilled with a laser engraver (VLS2.30, Universal Laser Systems). The two cover glass slides and one (100 μm) or two (200 μm) layers of a paraffin wax film (Parafilm M, Bernis USA) were sandwiched and heated up for a few seconds at 120 °C to assemble the flow cell. The cells were then incubated with an Antidigoxigenin (25 ng μl^–1^) solution (Roche) overnight at 4 °C and were passivated for at least 2 hours using BSA (NEB). The cells were stored in a humid and sealed container at 4 °C until further use.

Capillaries were cleaned, functionalized and passivated using the same procedure as described for cover glass cells. PFTE tubing for buffer and sample introduction was attached to capillaries using thermo retractile tubing.

### 4.4. Fabrication of *λ*/2 DNA

*λ*/2 DNA molecules were fabricated based on a previously published method.^54^ Briefly, CosR-tail and CosL-tail oligonucleotides (see Table S3†) were biotin tailed and the XbaI-A oligonucleotide was digoxigenin tailed using Terminal Transferase (NEB) and either BIO-dUTP or DIG-dUTP (Roche). The modified oligonucleotides were purified using a Qiaquick nucleotide removal kit (Qiagen). N6-Mehtyladenine free *λ* DNA (NEB) was cleaved with XbaI, giving two 24 508 bp fragments. These fragments and the three tailed oligonucleotides in addition to the XbaI-B oligonucleotide were subsequently annealed and ligated overnight using T4 DNA ligase (NEB).

### 4.5. Magnetic tweezers experiments

Tethers of *λ*/2 DNA molecules were obtained by mixing the DNA sample with streptavidin coated superparamagnetic 1 μm or 2.8 μm sized beads (Dynabeads, MyOne streptavidin, Invitrogen) in a buffer containing 10 mM PB (pH 7), 10 mM NaN_3_, 0.2 mg ml^–1^ BSA, and 0.1% Tween 20. DNA-bound beads were introduced in the flow cell and incubated for 10 minutes. Then the magnets were approached at a force of 4 pN to release non-specifically bound beads. Unbound beads were further washed using the same buffer. The zero extension of DNA tethers was determined by releasing the magnet. For the lateral pulling experiments, the *xy* center of the bead was determined by introducing rotations.

Operation of the vertical magnet, bead tracking and subsequent force analysis were performed using custom software written in LabVIEW 2011 (National Instruments), which incorporates corrections for blurring and aliasing.^38^,^39^ Nevertheless, these effects are negligible considering the length of the DNA and the small applied forces. The lateral magnet was controlled by using the commercial software Motion System 2.0 (PiezoMotor). All the experiments were performed at an acquisition frequency of 120 Hz.

### 4.6. Flow stretch experiments

Tethers of *λ*/2 DNA molecules were obtained by mixing the DNA sample with 1 μm sized streptavidin coated superparamagnetic beads (Dynabeads, MyOne streptavidin, Invitrogen) in the same buffer and under the same incubation conditions used for magnet calibrations.

Before flow-stretching the molecules, a force–extension curve was measured for each of them, and the anchoring point of the bead was determined introducing rotations. Tracking and offline data processing were carried out using custom written software in Labview 2011. All the experiments were performed at an acquisition frequency of 120 Hz.

### 4.7. ParB experiments

Tethers of *λ*/2 DNA molecules were produced in a buffer containing 100 mM NaCl, 50 mM Tris (pH 7.5), 4 mM MgCl_2_, 0.2 mg ml^–1^ BSA, and 0.1% Tween 20 (ParB reaction buffer). DNA molecules were laterally stretched at non-permissive forces for condensation over 1–2 pN. Then, 500 nM ParB^AF^ was injected into the cell and the DNA molecules were imaged using Andor Solis software. Images were acquired at a frequency of 9.52 Hz, using the EM level of 100 and cooling the sensor to –80 °C. Laser power was set to 1 mW. For condensation experiments, the lateral magnet was moved away from the flow cell to apply a force of 0.3 pN, while recording the fluorescence image. Fluorescence data analysis and kymographs were generated using ImageJ.^55^

## Conflicts of interest

There are no conflicts to declare.

## Supplementary Material

Supplementary information
