# Supplementary material for: Force determination in lateral magnetic tweezers combined with TIRF microscopy
Source: Nanoscale. 2018 Feb 20;10(9):4579–90. doi: 10.1039/c7nr07344e (PMC5831119; doi:10.1039/c7nr07344e)
Supplement: Supplementary file 1 [file NR-010-C7NR07344E-s001.pdf]

Electronic Supplementary Material (ESI) for Nanoscale.
This journal is © The Royal Society of Chemistry 2017

Supplementary Information

Force determination in Lateral Magnetic Tweezers combined with TIRF microscopy

J. Madariaga-Marcos[a], S. Hormeño[a], C. L. Pastrana[a], G. L. M. Fisher[b], M. S. Dillingham[b], and F. Moreno-Herrero *[a]

[a]Department of Macromolecular Structures, Centro Nacional de Biotecnología, Consejo Superior de Investigaciones Científicas, 28049 Cantoblanco, Madrid, Spain
[b]DNA:Protein Interactions Unit. School of Biochemistry, Biomedical Sciences Building, University of Bristol, Bristol, BS8 1TD, UK.

Calculation of extension for off-center attached beads

Vertical Magnetic Tweezers configuration (Fig. S6a)

$$z_{corr} = R(1 - \cos\beta) \tag{S1}$$

$$\beta = \sin^{-1}(r/R) \tag{S2}$$

$$l = z + z_{corr} \tag{S3}$$

where r is the distance from the bead axis to the DNA attachment point and can be determined by rotating the bead and fitting a circle to the xy positions, R is the bead radius, and β the angle formed by the DNA attachment point and the horizontal plane (Fig. S6A).

Lateral Magnetic Tweezers configuration (Fig. S6B)

$$x^{**} = x - (R - z_{corr})\cos\alpha \tag{S4}$$

$$z^{**} = z + R\,(1 - \sin\alpha) + z_{corr}\sin\alpha \tag{S5}$$

$$l = \sqrt{x^{**2} + z^{**2}} \tag{S6}$$

In the capillary case α=0, and assuming the perfect horizontal geometry of the DNA (Fig. 3), $z^{**} = 0$ and Eq. S4 becomes

$$l = x^{**} = x - R + z_{corr} \qquad \text{(S7)}$$

Note that z_{corr}=0 for all the different configurations recovers the simplified scenario with the DNA molecule attached at the central axis of the bead at its lowest point.

Additional calculations for bead-based laminar flow experiments

Maximum Reynolds number in flow-stretch experiments:

The Reynolds number of the system is calculated as:

$$Re = \frac{2r\rho\, v_{mean}}{\eta} \qquad \text{(S8)}$$

where v_{mean} is the linear velocity corresponding to the applied flow ($v_{mean} \approx$ 3·10^{-3} m s^{-1} for the maximum flow rate), η the dynamic viscosity of the fluid (10^{-3} Pa s) and ρ the density of the fluid (1 g cm^{-3}). As the theoretical calculation is done in a circular tube, r is the equivalent radius for circular geometry, $r = (d \cdot w)/(d + w)$. In this case, $d \approx$ 200 µm and $w \approx$ 7 mm and we obtain a radius $r \approx$ 190 µm. This gives a Reynolds number of $Re \approx$ 0.001 << 2000. Thus our system is always under laminar flow conditions.

Mean theoretical velocity in the center of the bead:

The average velocity along the diameter of the bead is computed integrating the profile as:

$$v_{flow} = \frac{1}{2R}\int_{z-R}^{z+R} v_{max}\left(1 - \frac{(r-z)^2}{r^2}\right) dz = -\frac{v_{max}\left(3z(z-2r) + R^2\right)}{3r^2}$$

In our case, $r \approx$ 190 µm (the previously obtained value for the equivalent radius), R = 0.5 µm and we have considered z = 1 µm (based on the average value of our experiments). This gives $v_{flow} = 0.0105 v_{max}$, so 1% of v_{max}.

Table S1. References to the components of the *Lateral Magnetic Tweezers Module*.

Description	Manufacturer	Reference	Comments
Piezoelectric translation motor	PiezoMotor	LL1011A	
Piezoelectric motor controller	PiezoMotor	PMD101	
Encoder	PiezoMotor	102822	
Translation stage	Newport	M-MR1.4	Need two units
Bracket to breadboard	Home made		See Fig. S1 for technical drawing
Coupling between stages	Home made		See Fig. S2 for technical drawing
Coupling to motor	Home made		See Fig. S3 for technical drawing

Table S2. References to the components of the *Capillary Holder and Rotation Module*.

Description	Manufacturer	Reference	Comments
Piezoelectric rotary motor	PiezoMotor	LR17	
Piezoelectric motor controller	PiezoMotor	PMD101	
Timing belt	RS	778-5039	One unit
Timing belt pulley	RS	778-4752	Two units
Ball bearing	RS	612-5745	Four units
Brass tube			1 mm diameter
Glass capillary	Vitrotubes (Vitrocom)	8320	0.2 mm x 0.2 mm cross section
Capillary holder	Home made		See Fig. S4 for technical drawing
Motor holder	Home made		See Fig. S5 for technical drawing

Table S3. Oligonucleotide sequences.

Name	Sequence	Comments
CosL-tail	[Phos]AGGTCGCCGCCCAAAAAAAAAAAA	To be labeled with Biotin
CosR-tail	[Phos]GGGCGGCGACCTAAAAAAAAAAAA	To be labeled with Biotin
XbaI-A	[Phos]CTAGACCCGGGCTCGAGGATCCCC	To be labeled with Digoxigenin
XbaI-B	GGGGATCCTCGAGCCCGGGT	Unlabeled

TableS4. Parameters used in flow-stretch experiments

Parameter	Value	Units	Description
η	10^{-3}	Pa s	Dynamic viscosity of the fluid
ρ	1	g cm^{-3}	Density of the fluid
d	200	µm	Heigth of the flow cell
w	7	mm	Width of flow cell
r	190	µm	Equivalent radius on circular pipe
R	0.5	µm	Radius of the bead

Bracket to breadboard Material: aluminium

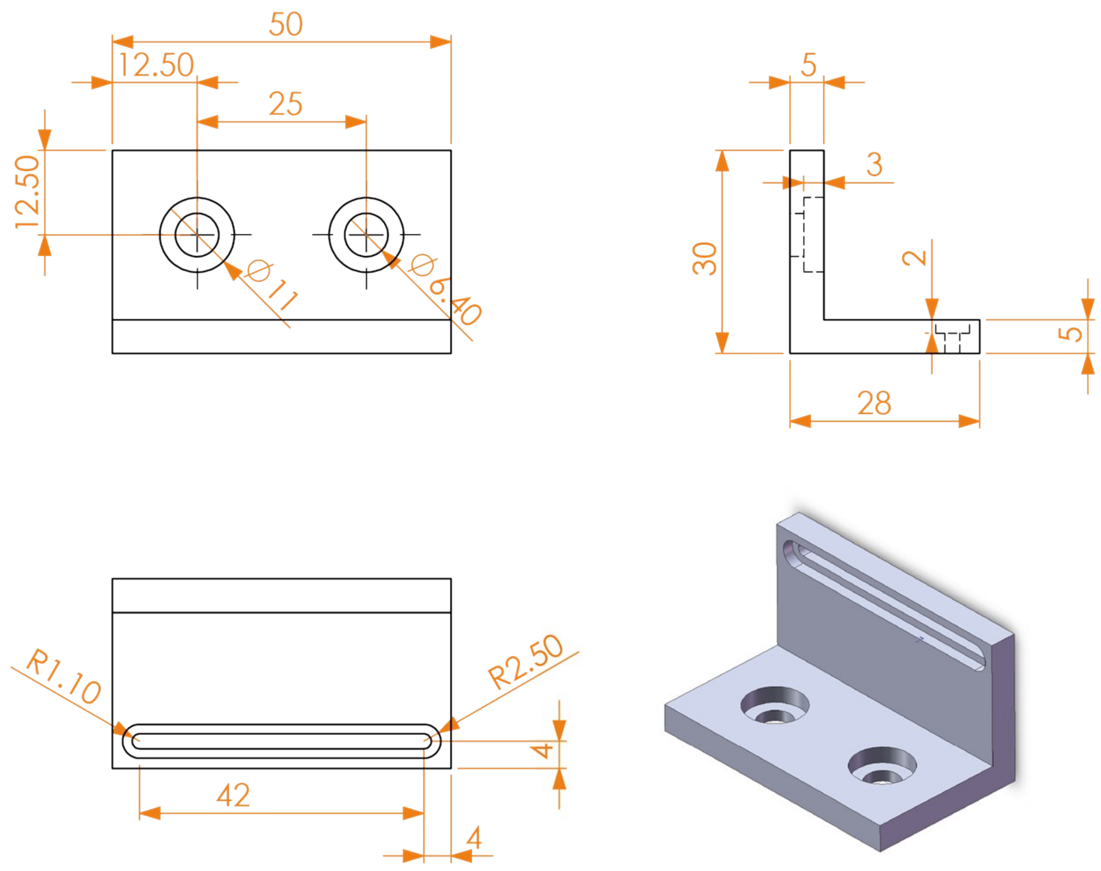

Fig. S1. Technical drawing of the "bracket to breadboard" component of the Lateral Pulling Module. All dimensions are in millimeters.

Coupling between stages   Material: aluminium

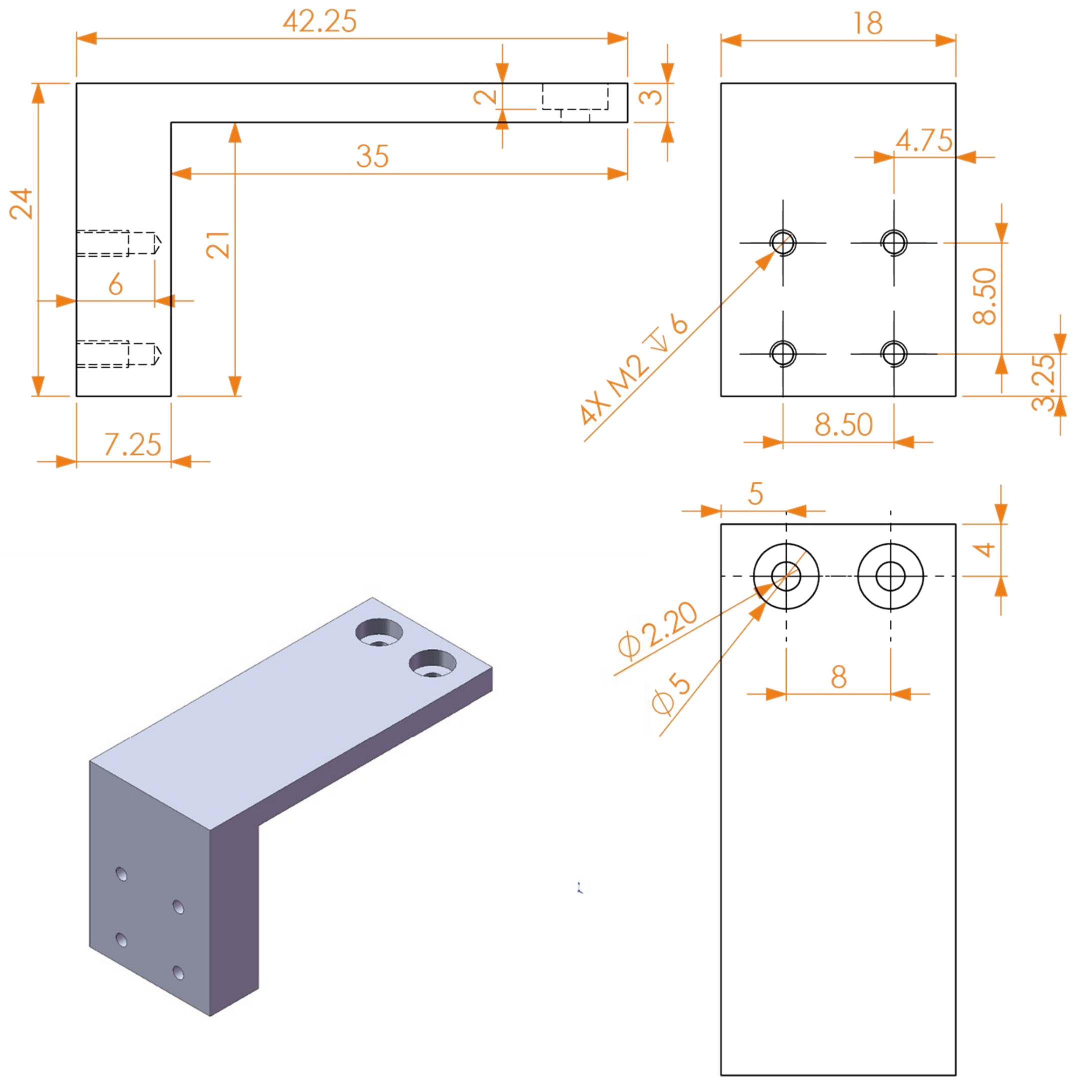

Fig. S2. Technical drawing of the "coupling between stages" component of the Lateral Pulling Module. All dimensions are in millimeters.

Coupling to motor   Material: aluminium

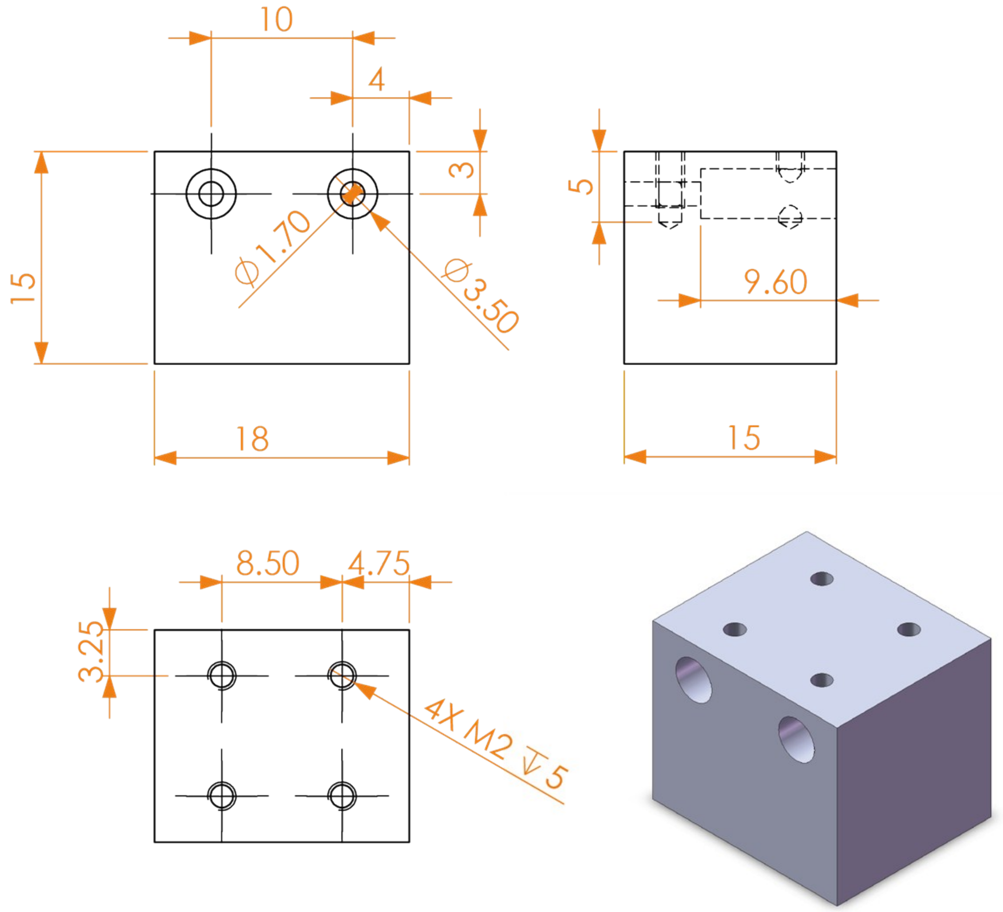

Fig. S3. Technical drawing of the "coupling to motor" component of the Lateral Pulling Module. All dimensions are in millimeters.

Capillary holder part A   Material: aluminium

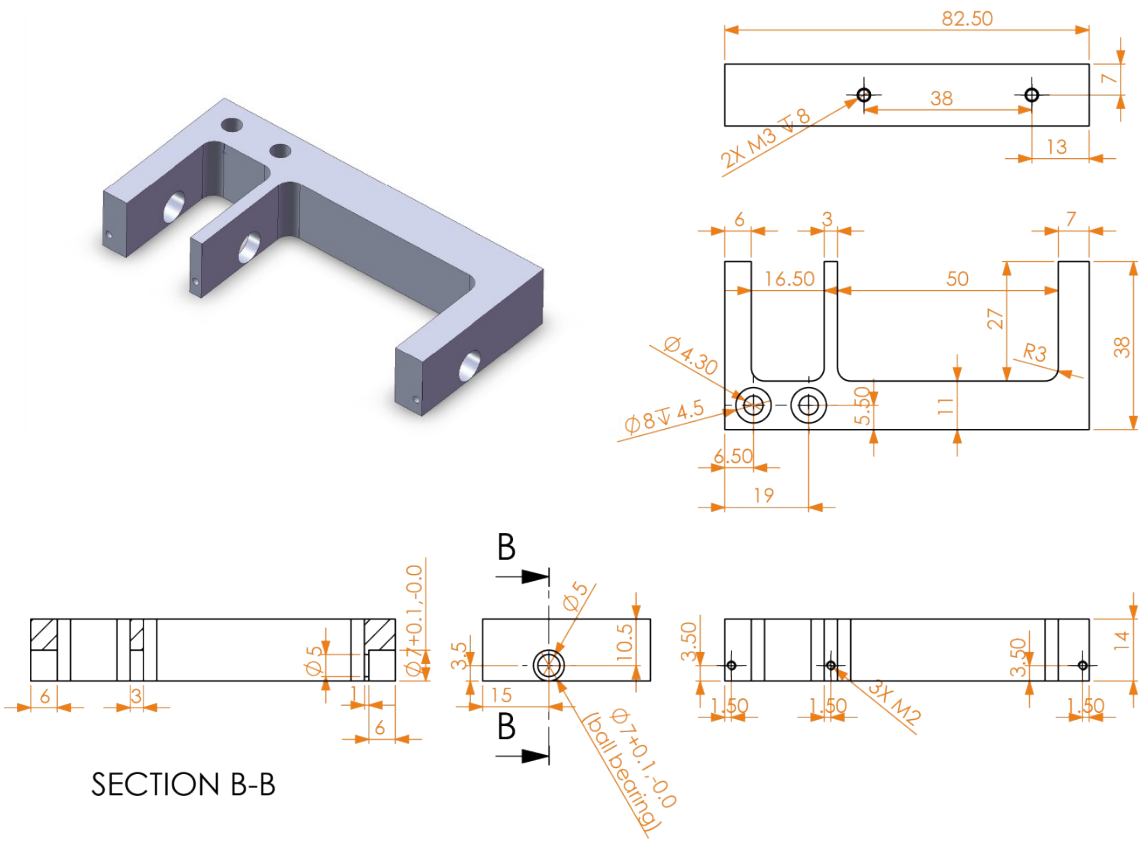

Fig. S4. Technical drawing of the "capillary holder" component of the Capillary holder and Rotation Module. All dimensions are in millimeters.

Capillary holder part B   Material: aluminium

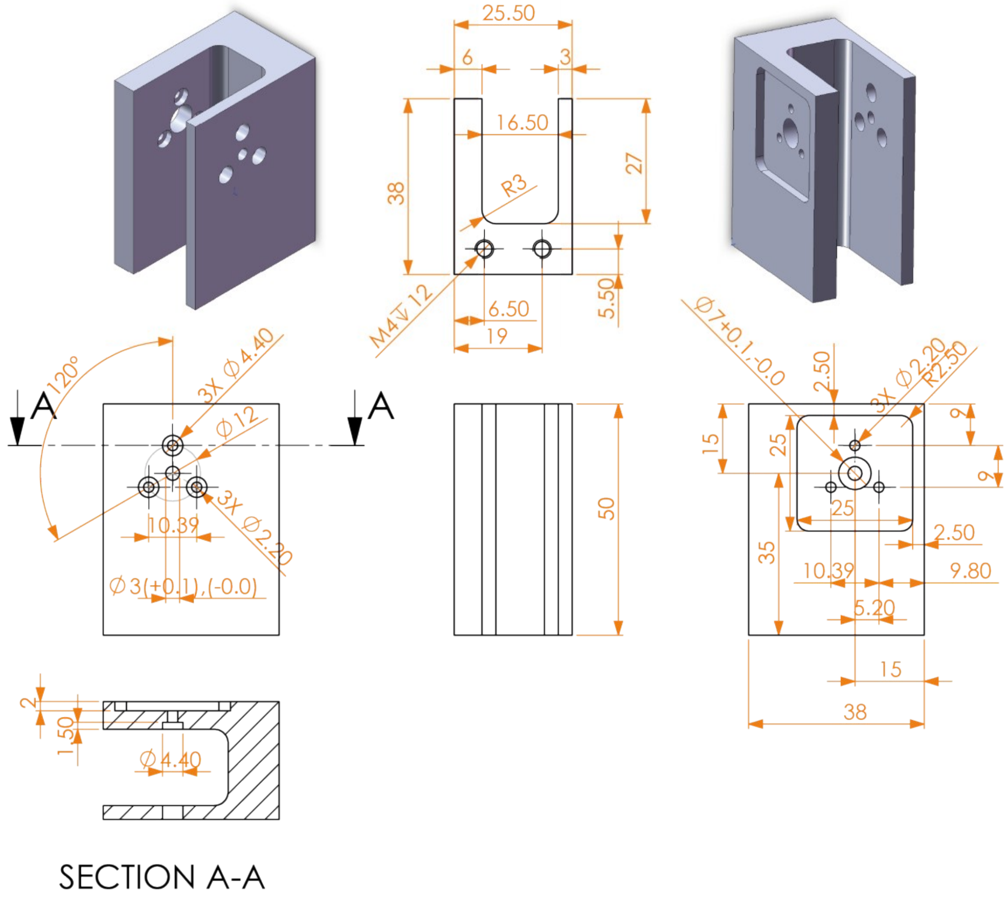

Fig. S5. Technical drawing of the "motor holder" component of the Capillary holder and Rotation Module. All dimensions are in millimeters.

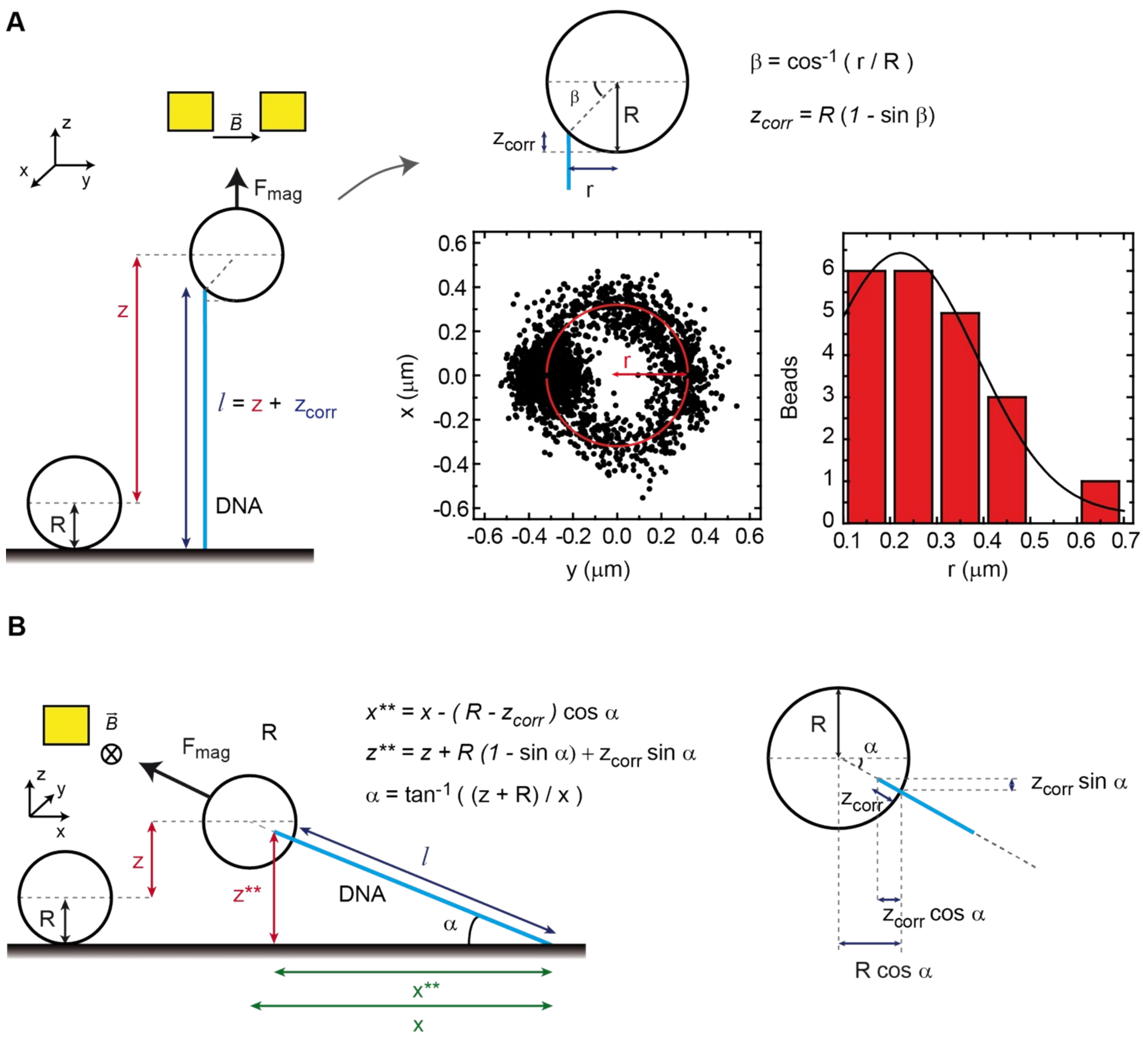

Fig. S6. Realistic measurement of the force in vertical and lateral magnetic tweezers in cover glass cells. (A) Cartoon of the geometric representation of extension measurements in vertical pulling, where the DNA molecule is anchored off-centre of the bead (left side). The extension needs to be corrected by a factor z_{corr} (right side, top), which can be obtained from the rotation radius (r) of the bead (right side, bottom). Histogram of rotation radius exhibits peak at 0.2 µm. (B) Cartoon of the geometric representation of extension measurements in lateral pulling, where the DNA molecule is anchored off-center of the bead. Extension is computed as $l = \sqrt{x^{**2} + z^{**2}}$, and includes a correction factor z_{corr} analogous to the vertical pulling case (detail in right side).

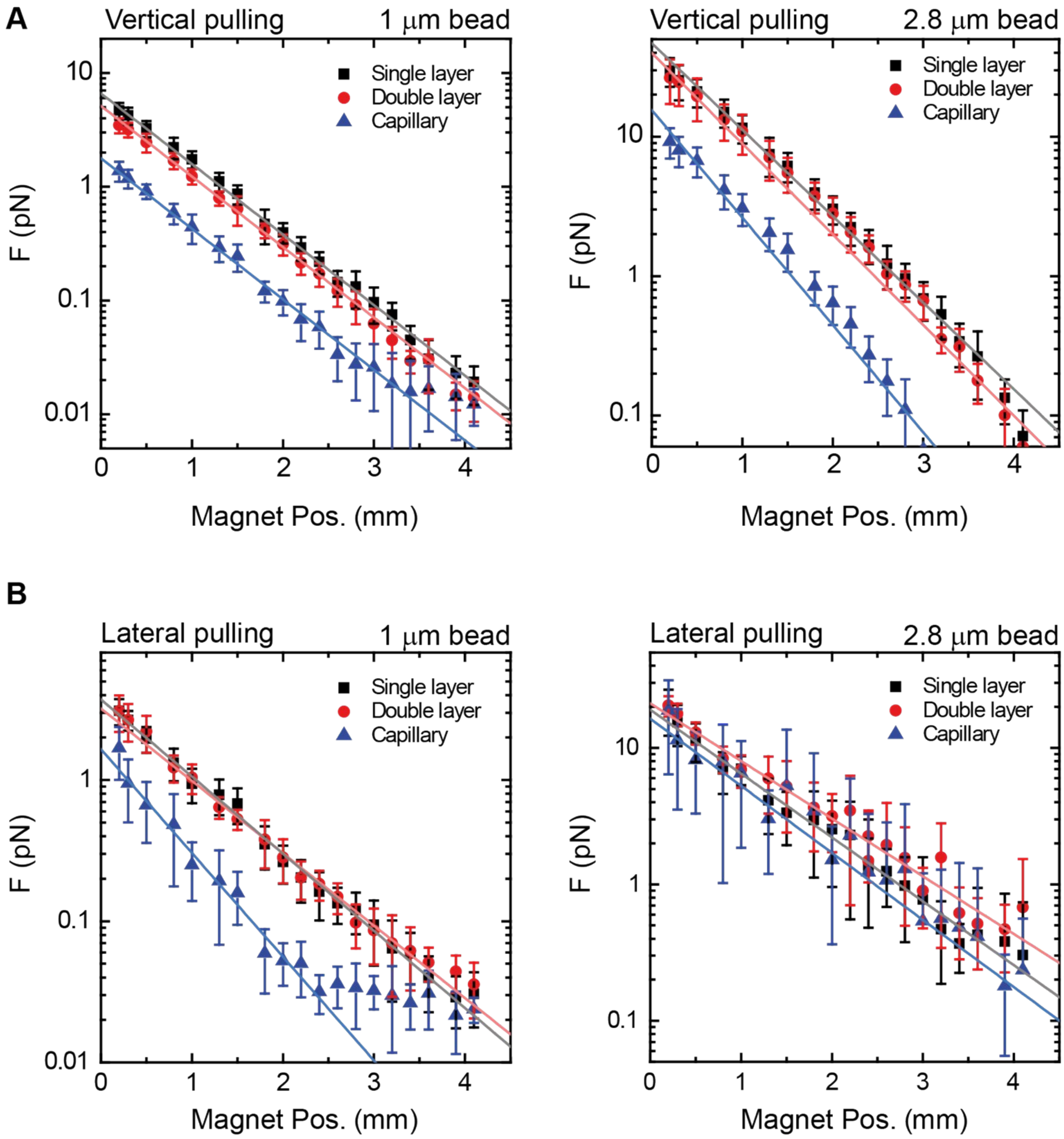

Fig. S7. Force as a function of magnet position for each magnet configuration and bead size. (A) Vertical magnets configuration and 1 μm beads (left). Lateral magnets configuration and 1 μm beads (right). (B) Vertical magnets configuration and 2.8 μm beads (left). Lateral magnets configuration and 2.8 μm beads (right). Data was obtained for λ/2 long DNA molecules and in flow cells of one or two layers of parafilm and in glass capillaries and then fit to an exponential function $f(x) = 10^{(-A \cdot x + B)}$ (solid line). Maximum forces are shown in Table 2. Error bars are the SD.

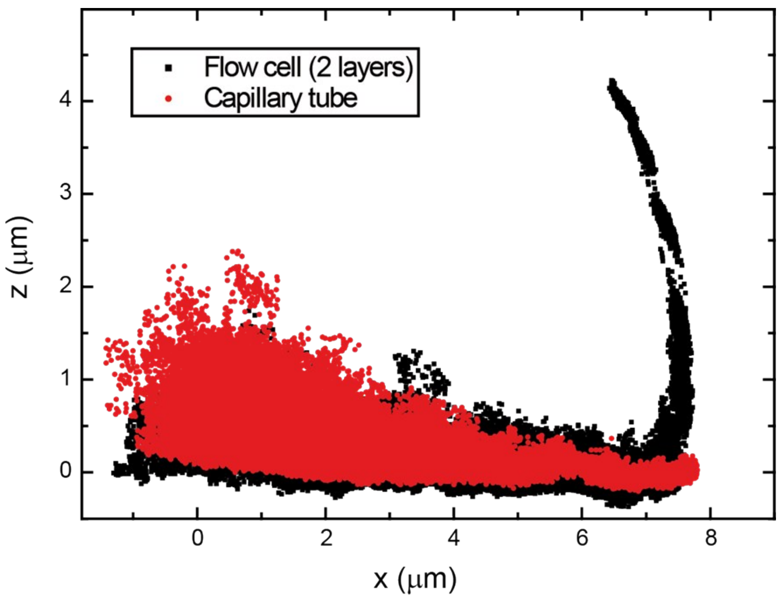

Fig. S8. Beads in regular two-parafilm layer flow cells show a lift-off upon the application of high forces. The same occurs in the case of single-layer cells (data not shown) but not in the capillary tubes, where the bead rests on the surface throughout the whole tracking.

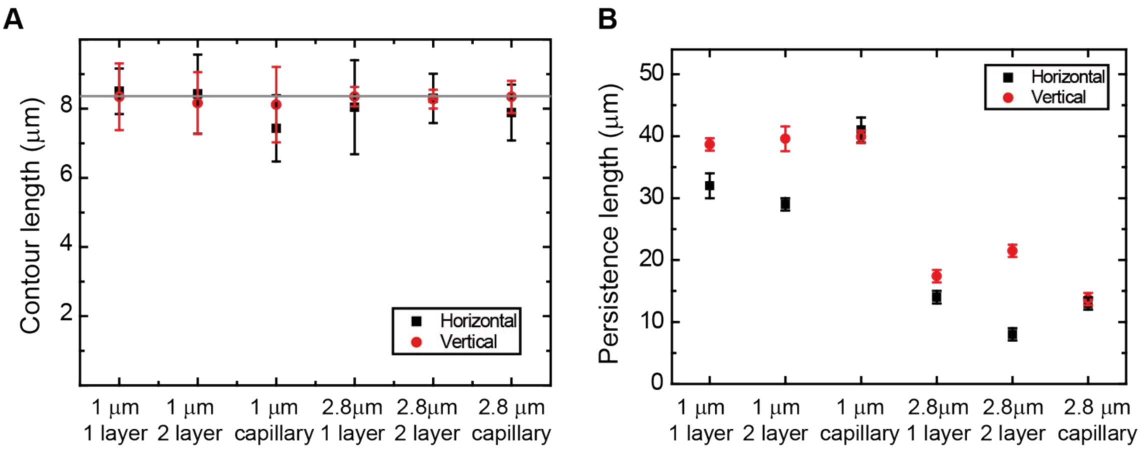

Fig. S9. Contour and persistence length for each magnet configuration and bead size. (A) Contour length values were obtained by fitting individual DNA molecules to the WLC model and then averaging them, errors are SD. The values agree with the theoretical length expected for a 24.5 kbp long DNA molecule. (B) Persistence length values were obtained by fitting an averaged force-extension curve to the WLC model in each of the conditions (errors are errors from the fit).

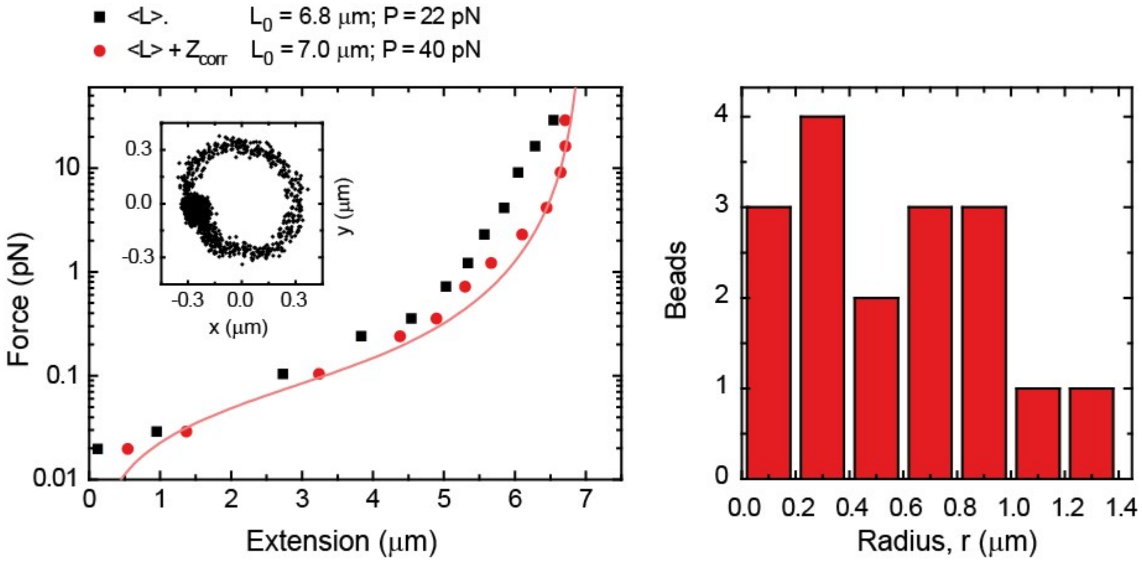

Fig. S10. The use of the correction factor z_{corr} considerably improved the fit to WLC model in off-center attached 2.8 μm beads in vertical pulling. [37] The rotation radius histogram in the case of 2.8 μm beads shows more dispersedly anchored beads than the one for 1 μm beads.

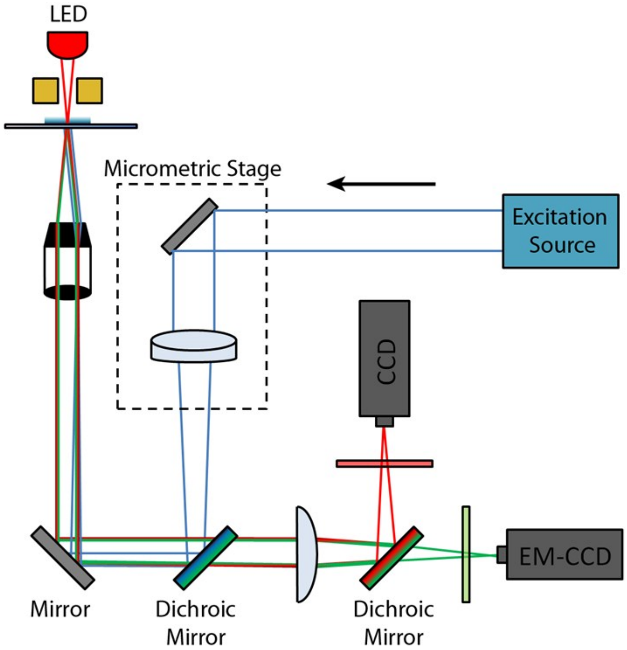

Fig. S11. Schematic home-built TIRF microscope setup. 488 nm laser light (Vortran Stradus) is reflected in a mirror placed in a micrometric stage which can be translated along the axis of incoming light, allowing to switch between TIRF and epi-illumination. A lens (Newport) focuses the beam onto the back focal plane of the objective (Olympus UAPON TIRF 100x). Light from the tweezers LED (Thorlabs) and emitted fluorescence is directed back to the objective, passed through a dichroic mirror and focused on an Andor Ixon Ultra 897 EM-CCD camera (for fluorescence) and Pulnix 6710CL CCD camera (for bright-field microscopy) by a tube lens (Newport). Another dichroic mirror allows the separation of both beams and the signals are subsequently filtered. All filters and dichroic mirrors on the fluorescence path are purchased from Chroma.
